# High fusibility and chimera prevalence in an invasive colonial ascidian

**DOI:** 10.1038/s41598-019-51950-y

**Published:** 2019-10-30

**Authors:** Maria Casso, Davide Tagliapietra, Xavier Turon, Marta Pascual

**Affiliations:** 1 Center for Advanced Studies of Blanes (CEAB, CSIC), Catalonia, Spain; 2 Department of Genetics, Microbiology and Statistics, and IRBio, University of Barcelona, Catalonia, Spain; 3 CNR - National Research Council of Italy, ISMAR - Institute of Marine Sciences, Venice, Italy

**Keywords:** Genetic variation, Cell biology, Whole genome amplification

## Abstract

The formation of chimeric entities through colony fusion has been hypothesized to favour colonisation success and resilience in modular organisms. In particular, it can play an important role in promoting the invasiveness of introduced species. We studied prevalence of chimerism and performed fusion experiments in Mediterranean populations of the worldwide invasive colonial ascidian *Didemnum vexillum*. We analysed single zooids by whole genome amplification and genotyping-by-sequencing and obtained genotypic information for more than 2,000 loci per individual. In the prevalence study, we analysed nine colonies and identified that 44% of them were chimeric, composed of 2–3 different genotypes. In the fusion experiment 15 intra- and 30 intercolony pairs were assayed but one or both fragments regressed and died in ~45% of the pairs. Among those that survived for the length of the experiment (30 d), 100% isogeneic and 31% allogeneic pairs fused. Fusion was unlinked to global genetic relatedness since the genetic distance between fused or non-fused intercolony pairs did not differ significantly. We could not detect any locus directly involved in allorecognition, but we cannot preclude the existence of a histocompatibility mechanism. We conclude that chimerism occurs frequently in *D*. *vexillum* and may be an important factor to enhance genetic diversity and promote its successful expansion.

## Introduction

Natural chimerism is a widely documented phenomenon occurring in multiple phyla of protists, fungi, plants and animals, including chordates such as ascidians and mammals^1^. Genetic heterogeneity within organisms represents an evolutionary challenge, as several potential risks and advantages varying among taxa have been suggested but rarely tested^2–4^. Among marine invertebrates, chimerism and allorecognition have been studied in the main groups with colonial or modular species: Cnidaria^5,6^, Tunicata^7^, Porifera^8,9^ and Bryozoa^10^. These organisms show highly polymorphic histocompatibility systems which determine the output of conspecific interactions^11,12^. Chimeras may last for the lifetime of the colony^13^ or only for a few days, depending on the compatibility of the contacted colonies^11^. Besides the increase of genetic variability, chimerism in marine invertebrates can provide multiple benefits (e.g. enhanced growth rates, reproduction, survivorship, competition and environmental tolerances) but also significant disadvantages (e.g. developmental instability, somatic and germ cell parasitism)^4,14^.

Within tunicates, chimera formation in botryllid ascidians is the most studied system, particularly in *Botryllus schlosseri*^15^. In this system, a single gene locus with multiple alleles determines the outcome of the colony contacts. Colonies are usually heterozygous at this locus and they fuse when sharing at least one allele^16^, which means fusion between colonies occurs when they are genetically similar. Following fusion, the somatic and germ-line components of the composite unit may compete with variable outcomes^17–20^. In *B*. *schlosseri* there is no evidence of an improvement in growth rates, reproduction or survivorship associated to chimerism, so other ecological or evolutionary advantages should favour chimerism in this species^21^. Botryllid ascidians possess a common vascular system that mediates the fusion/rejection outcomes of intercolony contacts. Other colonial ascidians (e.g., didemnids), however, lack colonial blood vessels. Without vascular connections, the scope for exchange of stem cells and cell lineage competition is greatly reduced, which seemingly reduces the potential for strict colony specificity and favours more indiscriminate fusion between colonies^22,23^.

In invasive populations where the low genetic diversity caused by the founder effect is initially a disadvantage^24^, chimerism may be boosted. Increased fusion rates among different colonies could result in higher genetic diversity and richer gene expression patterns, promoting the invasiveness of the species and turning the disadvantage into an advantage for the founder population^13^. Moreover, each genotype from a chimeric colonial individual may adapt better to different conditions in changing environments, enhancing colony survival^9,19^.

The colonial ascidian *Didemnum vexillum* is a worldwide invasive species that has colonized most temperate regions (see^25^, and references therein). It can form large colonies on either natural or artificial substrates, and it can overgrow other invertebrate species such as commercial bivalves in aquaculture facilities, causing important ecological and economic loses^26–28^. *D*. *vexillum* can form chimeric colonies^29^ and it can also reproduce asexually by natural or human-mediated fragmentation, which is probably a major enhancer of its spread^26^. Its reattachment capability and fragment viability contribute to its invasive success^29–31^. Chimerism has also been suggested as a driving mechanism of the species’ remarkable invasiveness^32,33^.

The study of chimerism in ascidians has relied on different techniques. Monitoring of colonies in the field allows the assessment of natural fusion rates^34–36^. Chimeras can also be induced experimentally by putting in contact colonies, either growing edges or cut surfaces^15,37^ and examining the outcomes in the laboratory. Field and laboratory studies should ideally be complemented with genetic analyses to demonstrate intracolony heterogeneity or to characterize interacting partners. Chimeric colonies can be detected by analysing different fragments of the same colony using several genetic techniques. One of the most used methods is microsatellite genotyping, applied to ascidians^38–42^, cnidarians^43^ and sponges^9^. This kind of studies may underestimate the prevalence of chimerism, as it can only be detected when more than 2 alleles are found at a given locus^38^. Other genetic markers used include Cytochrome Oxidase subunit I sequence data^44,45^ or randomly amplified polymorphic DNA–PCR (RAPD–PCR) band patterns^46^. The detection of chimeric individuals may be improved with more markers, and whole-genome scanning techniques such as genotyping-by-sequencing (GBS) generate large amounts of genetic markers which can be applied to non model organisms^47^. In samples with scarce DNA material, a whole genome amplification (WGA) step is needed to obtain enough DNA^48^. The combination of WGA and GBS has been shown to reliably estimate multilocus genotypes in *D*. *vexillum* for clone detection and population genomics^25^ and can be an efficient and precise tool to assess chimerism.

In this study, we assess chimerism in *D*. *vexillum* and combine field surveys and experimental fusion tests with WGA-GBS genomic analyses from single zooids. Our objectives are (i) to report the prevalence of chimeric colonies in an introduced locality, (ii) describe the fusion/rejection behaviour between colony pairs, (iii) analyse the relation between colony fusion capability and genetic distance genomewide, and (iv) scan the dataset for candidate loci mediating colony fusion.

## Methods

Two different approaches were followed to study the chimerism in *Didemnum vexillum*: (a) the identification of chimeric individuals in the wild, and (b) fusion experiments.

### Sampling to identify chimeric individuals in the wild

**Figure 1:**
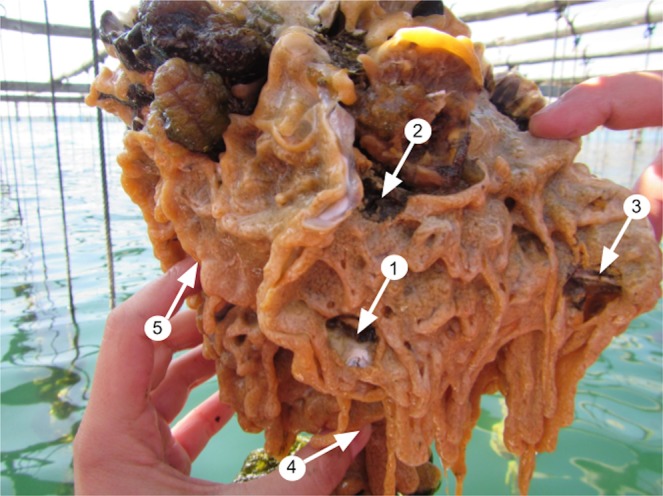
Sampling scheme of a colony of *D*. *vexillum* growing over commercial oysters in the Ebro Delta for the prevalence study. Arrows indicate the 5 sampled fragments: one central fragment (1) and four peripheral fragments (2–5). Fragments 4 and 5 are next to fingers and its position is not visible due to the 3D structure of the colony.

The first approach was carried out in oyster aquaculture facilities at the Fangar Bay (Ebro Delta, Spain, 40.776N, 0.737E). This system represents a favourable environment for *D*. *vexillum* in an enclosed area^49^,^50^. Nine colonies growing on commercial oysters were sampled. One central fragment and four peripheral fragments of 1 cm^2^, separated each other by at least 5 cm, were cut from each colony to determine the prevalence of chimeric colonies in this population (Fig. 1). The 45 fragments (5 for each of 9 colonies) were preserved in 96% ethanol for DNA extraction of a single zooid each.

### Sampling and fusion experiments

**Figure 2:**
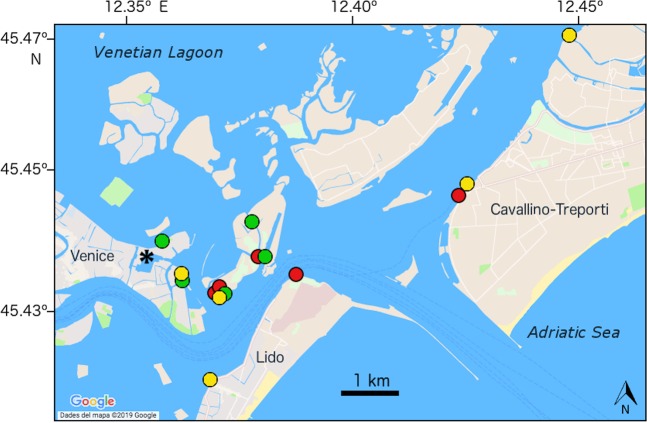
Sampling map of the 15 colonies used in the fusion experiment carried out in the Venetian Lagoon. Each colour represents a different set. The scale bar represents 1 km. The asterisk identifies the spot in the Arsenale where the slides for the fusion experiment were submerged. The map was obtained and modified from Google Maps.

In the second approach, a colony fusion experiment was carried out at the Venetian Lagoon with 3 sets of 5 colonies each (Fig. 2). This location is well suited for experimental work because it can be accessed directly from the laboratory facilities of the Institute of Marine Sciences (CNR-ISMAR). From each colony, identified with letters from K to Y, a fragment of <20 cm^2^ was cut into 7 pieces of 1–2 cm^2^ with a scalpel. One fragment was preserved in 96% ethanol for DNA extraction, two were paired with each other (intracolony pair), and the other 4 were paired to another colony fragment of the same set (intercolony pairs). The fragments used in this experiment were peripheral whenever possible, as it is described that they reattach faster than central fragments^29^. In total, there were 45 pairs, corresponding to 5 intracolony and 10 intercolony pairs for each set. All manipulation was done in the laboratory within hours of collection, and taking care to keep the colonies submerged in freshly collected lagoon water at all times.

Each colony pair was fixed on a glass slide using cotton threads, with contacting cut edges to trigger a fast fusion/non-fusion outcome. The slides were submerged in vertical position at 2 meters depth at the ISMAR docks located in the ancient harbour of the Arsenal of Venice (Fig. 2). The study was carried out for 30 days, within the May-June period, coinciding with the growing season of *D*. *vexillum* in the region^51^. The slides were photographed twice a week to track colonies’ growth and fusion behaviour.

### DNA extraction, sequencing, and loci identification

A thorax of a single zooid from each sample was dissected under the binocular and used for DNA extraction. We used an individual zooid to make sure we had a genetically homogeneous unit, and selected the thorax to avoid contaminating DNA from gut contents and to prevent a mixture of somatic and reproductive tissues. To get enough DNA from single thoraxes, a whole genome amplification (WGA) procedure was carried out with the REPLI-g® Single Cell kit (Qiagen) following manufacturer’s protocol except for the use of 1.5 µL of polymerase instead of the recommended 2 µL^25^. All samples were sent to the National Center of Genomic Analysis (CNAG, Barcelona) where a genotyping-by-sequencing (GBS) protocol was carried out. DNA was digested by PstI restriction enzyme and a paired-end sequencing of 2 × 125 bp fragments was performed in an Illumina HiSeq2500 platform.

Sequence quality filtering and loci identification and selection was carried out using the GIbPSs toolkit^52^ and following the same pipeline described in a previous study^25^. In short, the sequence quality filtering included the elimination of lower quality last bases by truncation and removal of reads with a low average Phred score threshold. The loci identification was divided in two steps: per sample and globally. First, sequences were analysed separately by sample, grouping identical reads into sequence variants and then into loci by pairwise comparisons. Second, a global locus and allele identification was performed to construct the loci dataset. The last main stage was the loci filtering where loci with alleles that could be indel variants, deeply sequenced loci and loci with more than two alleles per sample were removed. After these filters, loci shared by less than 70% of the samples were also deleted. The samples of Ebro Delta and Venice were analyzed separately to get two final loci datasets since they are significantly genetically differentiated populations^25^.

### Data analysis

For each loci dataset, a table of haplotypic genotypes (i.e. alleles defined combining all variable positions of each locus) and a genepop file were exported from GIbPSs. The table of genotypes was used to get the Percentage of Shared Genotypes (PSG), defined as the percentage of identical genotypes among shared loci^25^. The PSG was calculated in R^53^, plotted using ‘ggplot2’^54^ and used to identify unique multilocus genotypes. Pairs of samples with PSG higher than 90% were considered the same genotype^25^. The genepop file was read into R using the ‘adegenet’ package^55,56^ and used to calculate the pairwise genetic Prevosti distance in ‘poppr’^57,58^ corrected by the exact number of loci shared by each pair.

The corrected Prevosti genetic distances between fused genotypes from the prevalence study and fused genotypes from the fusion experiment were compared with a Mann-Whitney-Wilcoxon test. For the fusion experiment, the corrected Prevosti distances between fused and non-fused intercolony pairs were compared with a Mann-Whitney-Wilcoxon test. The time to fuse between fused intercolony pairs and fused intracolony pairs was also compared with a Mann-Whitney-Wilcoxon test. A Pearson correlation coefficient between time to fuse and genetic distance was calculated for all fused pairs. All the analyses were run using R^53^.

For each locus of the fusion experiment dataset with less than 20% of missing data, the number of shared alleles (ranging from 0 to 2) of each surviving intercolony pair was calculated. Among them, we selected 1% of the loci with the highest absolute difference in shared alleles between fused and non-fused pairs. Consensus sequences of each selected locus were aligned using Blastn searches to the genomes of *Ciona intestinalis* (INSDC Assembly GCA_000224145.1; KH, Apr 2011) and *C*. *savignyi* (CSAV 2.0, Oct 2005) available on the Ensembl genome database^59^ (http://www.ensembl.org/index.html). Only hits with an E-value below 10^–2^ were considered.

## Results

### Prevalence study

An average of 2,510,418 raw reads per sample were obtained in the prevalence study at the Ebro Delta and 79,1% remained after the sequence quality filtering stage. A total of 69,600 loci were found among the 45 samples of which 2,145 polymorphic loci with 6,602 alleles were shared by at least 70% of the samples (Supplementary Dataset S1). The number of loci per sample averaged 1,995 but one sample shared only 536 loci (25% of the total loci), and was removed for further analyses.

**Figure 3:**
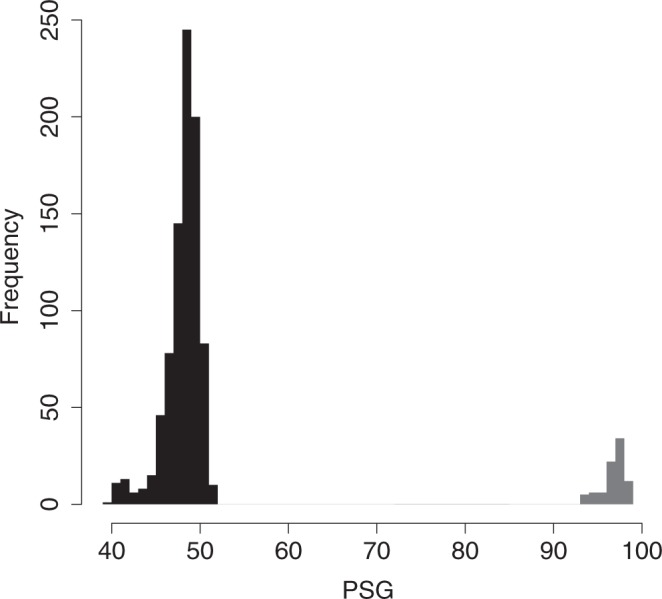
Frequency distribution of percentage of shared genotypes (PSG) values. PSG values between samples with different genotypes are in black and PSG values between samples with the same genotype are in grey.

PSG clearly identified pairs of samples with the same genotype, which had identical alleles in 96.83 ± 0.14% (mean ± SE) of the loci, and pairs of samples with different genotypes (48.07 ± 0.07%) (Fig. 3). Based on these differences, we identified 13 unique genotypes among the 44 assayed zooids from the 9 colonies sampled at the Ebro Delta: 3 colonies showed 1 genotype, 3 colonies had 2 genotypes and 1 colony had 3 different genotypes. Additionally, 2 colonies presented the same genotype and were therefore identified as clones. Thus, 4 out of 9 sampled colonies were chimeric which implies that in the introduced population of the Ebro Delta we found 44% prevalence of chimerism. The mean corrected Prevosti genetic distance among genotypes in the chimeric colonies was 0.216 (SE ± 0.005).

### Fusion experiment

We obtained an average of 2,127,134 raw reads per sample in the fusion experiment at the Venice lagoon and, after the quality filtering stage, 77.9% sequences were retained. We found a total of 44,688 loci of which 2,597 were polymorphic and shared by at least 70% of the samples (Supplementary Dataset S2). The total number of alleles was 7,343 and the mean number of loci per sample was 2,362. PSG values between colonies averaged 46.29% (SE ± 0.24), thus each sample had a distinct genotype and no clones were detected.

**Figure 4:**
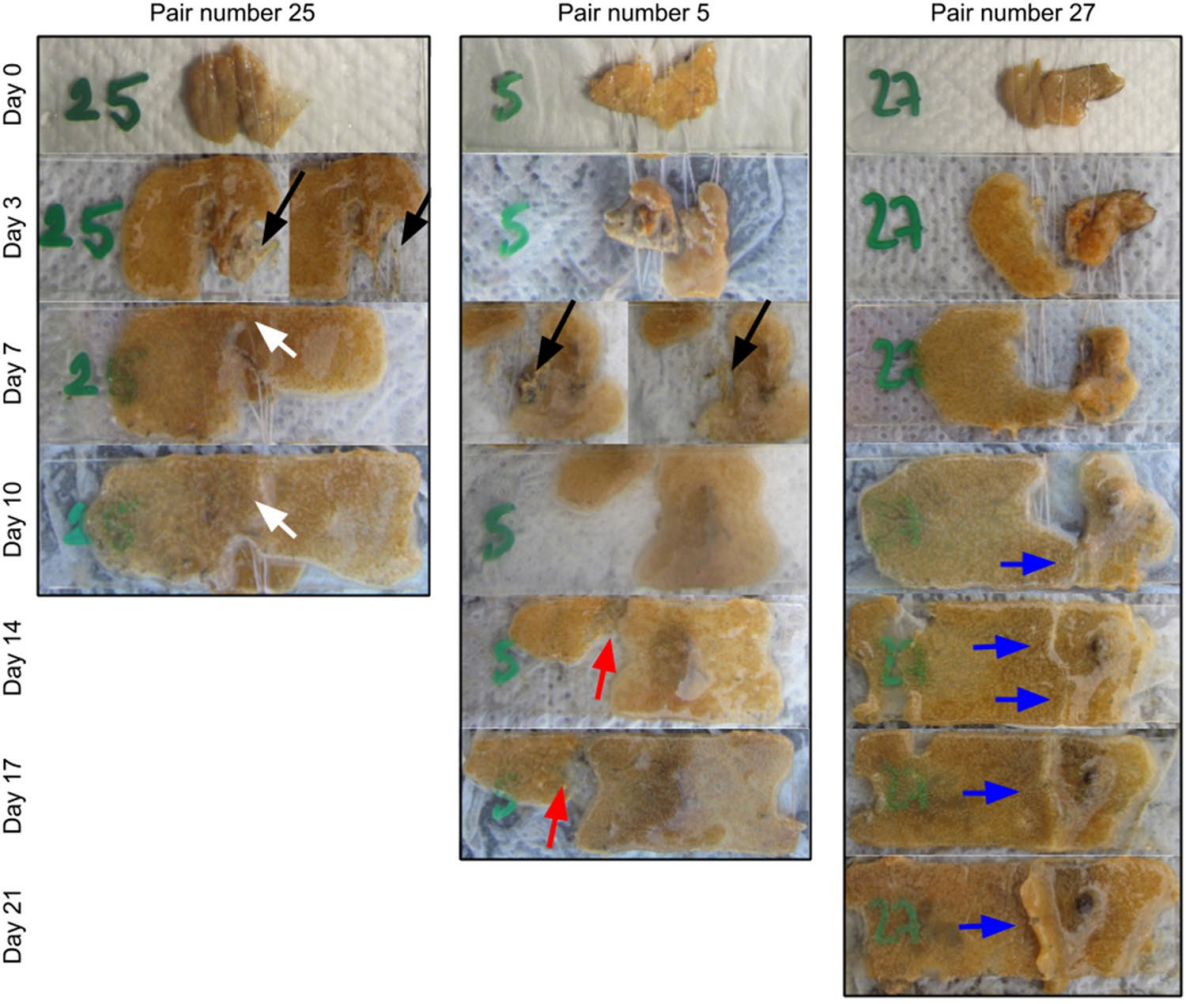
Progression of 3 representative pairs of colonies during the fusion experiment showing the different behaviours observed. Pair number 25 (two fragments of colony R) corresponds to a fused intracolony pair, and numbers 5 (colonies K and O) and 27 (colonies R and T) are non-fused intercolony pairs. Black arrows indicate dead sections (before and after removal). White arrows show a complete fusion. Red arrows point out rejection with fragment retreat outcome. Blue arrows indicate non-fusion front formation. All photos are scaled and cropped to fit approximately the supporting slides, which are 25 × 75 mm. Photographic sequences are selected to illustrate the variety of possible outcomes, however, the experiments continued and lasted for 30 days.

After the experiment set up, although the fragments were placed with contacting cut surfaces, most of them had to reattach, seal the cut, and grow into contact again. Some fragments reattached completely, while others only reattached partially, producing dead sections (Fig. 4). These dead sections were carefully removed as soon as they were detected. However, from the total of 45 pairs (15 intra- and 30 intercolony pairs), one or both fragments from 20 pairs (7 intra- and 13 intercolony pairs) could not reattach to the slide and thus died before any contact. No specific fragment typology (i.e. growing edge or central fragment) nor other external characteristics (i.e. colouring, thickness, roughness) seemed to correlate with mortality. All 8 surviving intracolony pairs and 5 intercolony pairs fused, while 11 intercolony pairs did not fuse. The number of fused, non-fused and dead pairs between the three different experimental sets were not significantly different (chi-squared = 2.65; p = 0.85).

We observed two different fusion phenotypes: complete fusion and partial fusion with some non-fusion front formation. All 8 surviving intracolony pairs fused completely (Fig. 4) and the limit between each fragment was rapidly blurred. On the contrary, only one out of the 5 intercolony pairs that fused did it completely and rapidly, while the others showed different behaviours. Two pairs fused only partially and formed a non-fusion front in a portion of the contacted edges. Another pair contacted at two separated points, in one of them colonies fused and in the other one of the colonies retreated. The last fused intercolony pair had a more complex behaviour, after one week of reattachment and growth, colonies met and fused at one contact point. However, a week later one of the colonies regressed partially at this point, while contact occurred again at a second point, where no fusion occurred. Finally, both colonies fused again at a third point of contact two weeks later.

We also observed two different non-fusion phenotypes: rejection with stable non-fusion front formation, and rejection with regression. All 11 non-fused colonies were intercolony pairs. Most of them (7), presented a non-fusion front all along the contacted region (Fig. 4). The other 4 pairs also formed an initial non-fusion front, only incipient in two of them, but one of the colonies finally retreated from the contact margin (Fig. 4).

**Figure 5:**
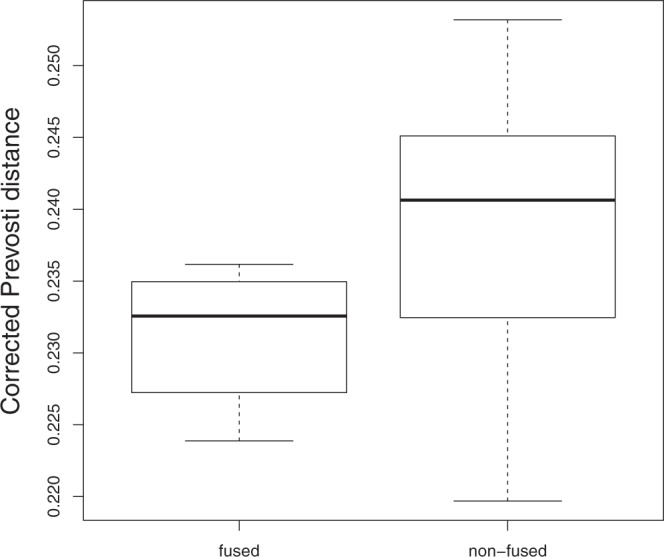
Corrected Prevosti distance between fused and non-fused intercolony pairs.

The comparison between the mean corrected Prevosti distances of fused intercolony pairs (0.231 ± 0.002) and non-fused intercolony pairs (0.239 ± 0.003) showed no significant difference (Mann-Whitney-Wilcoxon = 42; p = 0.110) (Fig. 5). The time from first day of contact to first day of fusion between intracolony (0 to 4 days) and intercolony pairs (0 to 6 days) was not significantly different either (Mann-Whitney-Wilcoxon = 29; p = 0.15). Likewise, the correlation between time (days) to fuse and Prevosti genetic distances was not significant (Pearson correlation = 0.44; p = 0.13). On the other hand, the difference in the mean corrected Prevosti genetic distances among fused pairs (0.231 ± 0.002) and among genotypes fused to form the detected chimeras in the prevalence study (0.216 ± 0.005) was marginally significant (Mann-Whitney-Wilcoxon = 4; p = 0.052).

### Candidate loci mediating colony fusion

**Figure 6:**
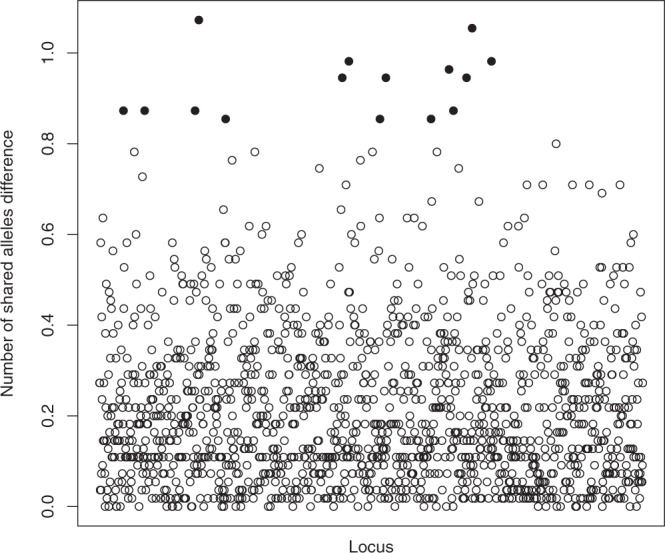
Absolute difference between mean number of shared alleles per locus among fused and non-fused intercolony pairs. Only loci with less than 20% of missing data are represented (N = 1,456). Solid dots correspond to the 1% loci with highest difference (>0.85). Loci are ordered by ID number in the x axis.

From the total 2,597 loci of the fusion experiment dataset, 1,456 showed less than 20% of missing data. A total of 15 loci (1%) were considered as possible candidate loci for mediating colony fusion (Fig. 6; Supplementary Dataset S3) based on having the highest absolute difference between the mean number of shared alleles of fused pairs and non-fused pairs. In all 15 loci, this difference was greater than 0.85 and positive indicating that more shared alleles were found in fused pairs.

Among the 15 Blastn searches performed against the *Ciona intestinalis* genome, we found 3 hits although with low query cover and identity values. One was aligned to an intron of a predicted gene corresponding to an uncharacterized protein from chromosome 2. Another was aligned to an exon of a predicted gene coding for an uncharacterized protein from chromosome 8. The third locus was aligned to an intron of the gonadotropin releasing hormone receptor 1 (*gnrhr1*) gene from chromosome 3 (Table 1). Among the 15 Blastn searches against *C*. *savignyi* genome, we found only 3 hits, with similar query cover and identity values as for *C. intestinalis*. Only one locus aligned to a gene region corresponding to an intron of a predicted gene (Table 1).

**Table 1:** Results of the Blastn search of each candidate locus on the genome of *Ciona intestinalis* (Ci) and *C*. *savignyi* (Cs).

Locus	Sp	Ch	Gene	Query cover %	E-val	%ID
9280	Ci	2	ENSCING00000000844	34.5	0.006	68.1
9280	Cs	unk	ENSCSAVG00000000305	33.0	0.003	69.7
27932	Ci	3	gnrhr1	23.5	0.006	76.6
36446	Cs	unk	none	26.5	0.0004	75.5
38223	Ci	8	ENSCING00000013148	24.5	0.0007	77.6
42739	Cs	unk	none	35.0	0.0003	70.0

Locus: ID number. Ch: chromosome number where the homologous fragment is located (unk: unknown). Gene: described or predicted gene. Query cover %. E-val: expected value. %ID: percentage of identity.

## Discussion

In colonial species, natural chimerism implies the presence of zooids with different genotypes within the same colony. We found a prevalence of 44% of chimeras in the *Didemnum vexillum* population of the Ebro Delta and 31% of intercolony pairs fused in the Venice experiment. The mean genetic distance between chimera-forming genotypes and experimentally fused colonies was 0.22 and 0.23 respectively. Both localities are heavily invaded by *D*. *vexillum*^50,51^, and the populations are genetically diverse and well differentiated^25^. In other species of tunicates the outcome of contacts between colonies is genetically regulated^60^. However, in our experiments, the genetic distances between fused genotypes were not significantly different than between non-fused genotypes, and we could not ascribe a clear role to the possible candidate loci detected. Therefore, in our genome-wide scan we could not identify the relevant loci for the allorecognition reaction between different colonies, if any.

Most of the genetic methods used for chimera detection underestimate the actual prevalence of chimerism due to the polymorphism of the markers used^13^. Next-generation sequencing (NGS) technologies such as GBS^47^ provide a tool to obtain large number of loci from species lacking a reference genome, producing reliable data to identify chimeras. In samples with scarce genetic material or species with reduced individual size, the combination of a WGA method with GBS proved robust and effective^25^ to obtain a large panel of loci that can be used to assess chimerism. In previous studies, the Percentage of Shared Genotypes (PSG) has been used to identify clones^25^ and in the present study it proved to be a fast and easy tool to detect chimeras. When zooids of the same genotype were compared, ca. 97% of the loci had the same genotypes and the 3% discrepancies can be explained by amplification and sequencing artifacts which could not be filtered during the bioinformatic analysis^61^. PSG dropped to less than 50% when colonies with different genotypes were compared. Note here that the relevant point is the existence of such a marked gap in the percentage of shared genotypes, as the precise mean PSG values may differ between studies depending on technical aspects (e.g., the number of markers, the stringency of the filters, or the artifacts during the process)^61^.

Natural chimerism has been found in several compound ascidians, but its prevalence varies considerably between and within species, from 0.5 to 39% in *Botryllus schlosseri*^38–40,42^, 1.9% in *Botrylloides nigrum*^45^, 1% in *Perophora japonica*^44^, from 3 to 61% in *Diplosoma listerianum*^23^, and from 17 to 48% in New Zealand populations of *D*. *vexillum*^62^. Our 44% prevalence of chimerism in the invasive population of the Ebro Delta is in the upper range of values found in other species, but low within those found in introduced populations of *D*. *vexillum*. In cut surface fusion experiments, an 80% of chimerism has been reported using colonies from the introduced area in New Zealand against only 27% in the native range^32^. Our experimental results in the Venetian Lagoon (31% fusion) are closer to the values found in the native range and far from those reported in New Zealand. This discrepancy may be explained by the different origin and genetic composition of the introduced populations in Europe and New Zealand^25^. More populations of this species should be studied to assess the extent of chimerism associated to introduced populations.

In species where a vascular system allowing an exchange of cells within colonies exists, a well-developed allorecognition system may reduce fitness costs of chimerism associated with somatic and germ-cell parasitism^17,18,46,63^. Didemnid ascidians such as *D*. *listerianum* and *D*. *vexillum* lack a colony-wide vascular system and zooids are only connected by the tunic, greatly decreasing the exchange of cells and hence the costs of chimerism. This type of colonial species may have a reduced or even absent allorecognition system, favouring more indiscriminate fusion between different colonies and making chimerism a more common condition^46^. Similarly, the relatedness of fused colonies also varies among species. In *Botryllus* spp. fusion can occur only between kin colonies^39^, while in *D*. *listerianum*^46^ and *D*.*vexillum* (present study) no genetic control mechanism has been detected and fusion takes place between non-related colonies. However, a study on *D*. *vexillum* described accumulation of diverse cell types in the tunic adjacent to allogeneic fusion areas, mostly phagocytes and morula cells^64^. Thus, it is likely that these cell types mediate the recognition reaction in this species, and that some limited exchange of tunic cells occurs between interacting colonies.

The most common outcomes described in fusion experiments among fragments of colonial species are fusion or rejection. However, more complex patterns have also been reported in most species. For instance, in the hydrozoan *Hydractinia symbiolongicarpus* four types of allorecognition phenotypes can be observed: fusion, rejection and two types of transitory fusion^11^. The stony coral *Stylophora pistillata* shows eight types of allorecognition reactions between kin colonies^65^. In the ascidians *Botryllus schlosseri* and *Diplosoma listerianum*, many different possible outcomes have been described^22,41,66^. In colonies of the ascidian *Trididemnum solidum* observed in natural environments, fusion has been observed after weeks or months of non-fused contacted margins from different colonies^34^. In a previous study on *D*. *vexillum*, highly dynamic interactions were found when allogeneic colonies came into contact^33^ and in many cases, fusion was followed by active growth away from fusion zones. The fragments resulting from this retreat showed predominantly segregated genotypes and chimeras were therefore transient in a scale of a few (10–12) days. In the present study, *D*. *vexillum* showed four different allorecognition phenotypes: complete fusion, partial fusion with some non-fusion front formation, rejection with stable non-fusion front formation, and rejection with regression. Some interactions were highly dynamic showing a combination of outcomes as the colonies contacted multiple times with a different outcome each time. However, fused colonies remained so until the end of the observation (30 days). Similarly, the finding of large chimeric colonies in the field (Ebro Delta) indicated that chimeras were not just transient, but stable entities. The external appearance of the colonies of *D*. *vexillum* and their behaviour in fusion experiments are different when comparing the photographic material from the present and previous studies^29,33^. We kept our experimental colonies in the natural environment of the lagoon while these previous studies were made under laboratory conditions. In the present work, colonies looked thicker and less transparent, suggesting that fragments may grow healthier under natural conditions.

Among our large loci dataset, none of the loci that we considered as possible candidates to mediate fusibility blasted to known genes with functions relevant for the allorecognition mechanisms. GBS produces huge numbers of loci randomly distributed through the genome of the target species^47^. Depending on the enzyme and species used, the studied loci may be differentially distributed among coding and non-coding regions^67^. For *D*. *vexillum* and using the *PstI* restriction enzyme, only 20% of the searched loci had a blast hit in *Ciona* genomes. Moreover, the query covers were small (~30%) and the E-value large suggesting that most of the analysed loci in this species will be located in non-coding regions and thus hard to identify in distant genomes. Thus, although we could not apparently detect any locus either directly involved in histocompatibility or linked to the relevant ones that could reveal a genetic control of the fusion/non fusion mechanism, we cannot discard that some of the identified loci are associated to highly specific regions mediating allorecognition. Moreover, the contrasting allorecognition phenotypes in fused isogeneic and allogeneic colonies (i.e., complete fusion in the former and complex, partial fusion patterns in the latter) might be indicative of a histocompatibility mechanism mediating fusibility in this species.

*D*. *vexillum* shows multiple advantageous biological traits that make it an aggressive invasive species that has colonized temperate regions worldwide. It has a rapid growth rate, produces large numbers of short-lived planktonic larvae and lacks significant predators (but see^68,69^). The importance of chimerism in adaptation and invasiveness can differ between species^13^. In the non-invasive but worldwide distributed populations of the branching coral *Pocillopora damicornis*, high levels of chimerism were found in extremely variable and highly impacted habitats^70^, suggesting chimerism may be involved in the success of adaptation of the species. In corals, chimerism has been suggested as an evolutionary mechanism of resilience and adaptation to global climate change impacts^6^. Similarly, chimerism has also been proposed as a driving factor in invasion success^13,44^. In colonial species with high growth-shrinkage dynamism and high fusion rates, chimerism may be a key aspect of the fragment dynamics and needs to be assessed for an efficient management^62^. Moreover, in the ascidian *B*. *schlosseri*, invasive populations seem to show higher levels of chimerism than in their native range^13^, and the same has been suggested for *D*. *vexillum*^32^. Chimeric colonies may show the ability to shift to advantageous genotypes in changing environments^4^. The invasive populations of *D*. *vexillum* assessed in the present study, the Ebro Delta and the Venetian Lagoon, show high levels of prevalence of chimerism and fusion rates, respectively. Thus, the role of chimerism in the success of the worldwide expansion of *D*. *vexillum* cannot be underestimated.

## Supplementary information

Supplementary dataset S1

Supplementary dataset S2

Supplementary dataset S3

## Data Availability

All genomic data generated or analysed during this study are included in this published article (Supplementary Datasets S1–S3). Photographic material for the colony fusion experiment is available from the corresponding author on reasonable request.
