# Supplementary material for: High fusibility and chimera prevalence in an invasive colonial ascidian
Source: Sci Rep. 2019 Oct 30;9:15673. doi: 10.1038/s41598-019-51950-y (PMC6821838; doi:10.1038/s41598-019-51950-y)
Supplement: Supplementary file 3 — Supplementary dataset S3 [file 41598_2019_51950_MOESM3_ESM.pdf]

High fusibility and chimera prevalence in an invasive colonial ascidian

Maria Casso, Davide Tagliapietra, Xavier Turon, Marta Pascual

Supplementary dataset S3: ID number and sequence in fasta format of the 15 candidate loci with higher absolute difference between mean number of shared alleles per locus among fused and non-fused intercolony pairs.

>2126

AAACASAACCTTATTCCTTTTTAAACCAGTGAAAAAAAYGATCTTTTTGTAATATTGCAAAATATCCTTCTCTG
TTTGCTTTATTGATTTTTTAATTGAATATCACGTCATKCTTATCRMACCACCTAYAAYTAAAATTGTTGGAATA
WAATTGGAATTTCGTACAAAATTTATGGTACTGTAAATTTTCGCTTTYTTCA

>3784

GAACTAAAAGCAAGAAAACGTTTTCTCTTTTTATTTGAGTTTGCAGTTGCTCAAAGTTAGGCGCATGAAAGA
GCAGGTAATCTTGAGTATTTTTGTCTTCAAGTTAGGCGCATGAAAGAGCAGGTAATCTTGAGTATTTTTGTC
TTCCATGACTCCAACGAGASCCGAGAGGCACTTTTTTGGCTCAGGAGTCAGTCATG

>8985

CTTAAAYGTTTGTAAAAACGCACGATATTGTAGTAATCAAAACAATTGGATAACAGCTATAAATKGAACTTC
GACGAAATTATACCTGTTTACTAAGCCAATTGTTGCAGGCTCTTGAAGTAAAAAAAATTTTCAACTTTGCTCA
CTTTTAGTTCTTCTGCTTCTAGTCCTAAACTTGTCTCTGGACGTTTTGTCAAGTT

>9280

ATTATAACGTCARGTGATAAATAATGAGAAAAACTTGGTCATYTTCCATTCTTTTGTATGAAAGATTTTCTTCT
GTGGGGTTGTGAATGTTGTTTTATTTTGAAAGATTTTCTTCTGTGGGGTTGTGAATGTTGTTTTATTTTAGAC
TTTGTTATAGGAACATAATAATAAACAGTAAATCAATCGGCAAGAAACGCAGG

>11438

GAAAATTTTGGTTAAACTTATTTTTGGTGTTTKYTTGCAAACCTATTAACCAAATTATGCAAAAAATTGCTGTC
AGAGATCATATTCCACGGAGTGAAAGCTGGTCGCAGAGCAAGATTTGTATGAAATTTGTTTTATTGTCCGAT
AGTGCAAAATGTATGCAYTATATTTTCACTTATATGTTTGAGATGTAAATGTTG

>23738

CCTGYACYACAAACCACAAATKCTATCGAGTACAGAAGTTCTAGCAGTTTCATATTCTTTGAGCAATCAAACA
CGGACTATAWTWTTATCTGGGCTTCATGAGCAATCAAACACGGACTATAWTWTTATCTGGGCTTCATGAA
TATTTTRAAATGAAATAGCGTTTTCATCTTCAATGTATCATAGTTTAGGATGACAGC

>24274

CCAAAAGAACTCTGGGAAGATCCAAATCAATGGAATCAATGGAAGWAGGTGCTTTCGTKGTCCAAGTTTG
ACCGGATGAAAGYACGACCAATTTAGCCGCAATCAATGGAATCAATGGAAGWAGGTGCTTTCGTKGTCCA
AGTTTGACCGGATGAAAGYACGACCAATTTAGCCGCAAGAGGGCGYGCGTCCAACAAATC

>27281

ACATCAACACCGCRTATTAAAAGACTACGGATTAGAAAATGAATTTAATAAAACCGAAACAACTTGATGCAA
AAAGCGTAGTATTCGAKTTTTGTAAAAACAAAAAGCGTAGTATTCGAKTTTTGTAAAAACGATAAAATGCTT
AAAAGAAAAATGTAAAAACTTGAAACCTCTATTCTTCTTCCAGTTTGTACCATCAA

>27932

TGGTCAATTTTATTAAGAAAATAAATAAATTCAAAACTAAGAGCAGCACGAAGAAAAACACTACGAAGACA
TCTATTTACTAACATCTAAGATTTCATAAACATCTAAGATTTCATAATYGCCAGCATTGTRGTAAGAGATTTCT
TGAAAGGAGAGCTGGTTTCTTTTGAACTTGATAACGTCAGACGGGCCAAAGCATT

>33463

CAATGCCGTCACCAGAGCAAGCGAAGTGAARGAAAACCAAGTGAAAGGAAGGARCACAARCATTTGATTG
ACCTTAGAGAGACATCGTCGTTTCCGAGTCAATCAAGGGCGCTCGTAGACATTTTTGCAACGGGCAAATGTT
TTTTGGATAAAAAGAAAAGTGAAACTGRTGCTTTTGAAAACGCTATCATTGCAACAAC

>35804

CCTTCCTGACAAAAACAAACAATGCTTTCGGYAAATAAGAGTCAAACCATTATCACAAGGTCCAAAATCCGA
GATGCTAACCGCTACAGCACGGCAACCTCAACCTGGTTCTGTTTGTAATTTACGGTACTAGTGTTTTCGATTT
TCCTGTTTCGGCCTGATAAAATAGAAAACYTTCCRTAAAATAAGATAGAAAACMT

>36446

TTGATTAGATAAAATGCAATCATAGATGTTGAACTTCAAAGACTTTTTTYTTGTTTCGTTTAAAAAACTTTGAT
TGATTAATTGCGTCAATTCACCGACAYAAATCATTTAGAGTATAATCCATTGTGAATCGCTTCGCGGCAAAA
ATTCATTGTTTGGCTTTGAGAGACACRAAATTCAATGCCTCAACTCGGTCTATG

>38223

AAACAACTATCACACAGCATCACACTACTTTATCACCAACACAAGATGTCAGCAGSGTCTCAACAGCAACCC
AAGAAACAACAACTAAACTCTTTTTGCAACATCAACCACACTTGATTTAACAAGACTTTTAACAACTGYAAGC
ATGAGTACAAAGTCAACATTACCACAGTTTATCACTGAAACAACAATGCCAACAA

>39173

GTAGCTGAGAAATGATCTTTTGTAATTGTAGCTCTTCTTAATCTTCAGGAGCCTTGAAGWACGTGCCATACG
CGGAAGATCAAGCCTTGTTTCGAACTCAACTTGAAAAGCAGATTTCGTTGTTTTGAAACAAACGACTTTACT
CGGGCGGAARCAAAAGGAKACTGTTTCAGTTGGGTTATTTTTTTGCCGAGTCGCCT

>42739

TTGATTTTRGCTCTTTCGATGTGGATGGTTCAGCTTCCATTATTAAATCAAAACTTCACTTCTCTACACTAGGC
TACTAAGCTACTTTAGAATCAATTCAAGTTAGATAATGAAAACAAAACAAACTAAACAAAAACTCAACTCAA
TGTAAAATCATACTGATAACAATAATGTAACTCCTAAKTCTTGAATCATATCYC
